# Letter from Prof. J. P. White

**Published:** 1851-06

**Authors:** James P. White


### EDITORIAL DEPARTMENT.

### *Letter from Prof. J. P. White.*

Since the original department of the present number was in type, we have received two letters from Prof. White. In view of the interest which we presume our readers will have in the perusal of them, we give the first letter insertion in this place, deferring the second until our next issue.

PARIS, April, 1851.

MY DEAR DOCTOR:—Among the many hospitals of Paris, there are few which offer greater attractions to the medical observer than *Hotel Dieu:* nor are there any which possess more historical interest. Here was the great field of labor for Desault and Dupuytren,— for Bichat and Moreau, whose writings and opinions are consulted and respected, as much as those that have emanated from any similar number of men since the days of Hippocrates. Here, at this time, is to be found the venerable Roux, who, though he has occupied his present position for more than forty years, is still active, and making perhaps more operations than any other living surgeon, continuing to contribute to the improvement of his favorite department, thoroughly posted in the novelties of the day; and, what is still more remarkable at his time of life, ready to examine, without prejudice, any suggestions from the junior members of the profession. He is still erect and active, having the largest service in Paris. Commencing his visits at the early hour of seven in the wards, he did not complete the last amputation, on Wednesday last, when I followed him, until half-past eleven, although constantly and actively occupied. I may mention, also, that he not only secures the arteries himself, after making his amputations, but he does so without the aid of glasses, and as expertly as one at thirty, although now not less than seventy years old. Some idea may be conceived of the immense number of operations which Dr. Roux has made, by mentioning one fact which I have from his own lips. In conversing with him a few days since, he remarked that he had operated for cataract more than seven thousand times.

At Hotel Dieu also, are to be found Louis and Chomel, who are regarded as among the best pathologists of the present day. Each have large services, making daily visits; and three times each week delivering lectures to such students as choose to profit by their instructions.

But I have been more interested in the operations and lectures of Dr. Jobert, (de Lambelle,) who is one of the surgeons at this hospital, than either of those already mentioned. Dr. Jobert is now forty-five or fifty years old, in full health and vigor, slightly above medium height and size, erect and well formed in person, with a fine countenance, high forehead, large Roman nose; and, altogether, his personal appearance is remarkably good. He moves about the various wards with rapidity, executing all his operations with great dexterity, and calling with a voice almost stentorian, upon the *internes* to bring such instruments, bandages, sponges, or other apparatus as he may require. He lectures fluently, and so distinctly as to be heard in the remotest parts of the room, articulating every syllable, and thus enabling any one at all familiar with the language to comprehend all he says. So far as I have seen, he is, in my opinion, the Napoleon of French surgery — a bold operator—very expert in his manipulations — prompt in decision, and not by any means deficient in the more intellectual requisites of a good surgeon.

The principal inducements with me in following M. Jobert, have been to witness his operations, and watch the progress of the cases, as well as hear his views, explained by himself, upon that opprobrium of the art, *vesico vaginal fistula*, and other kindred affections. Perhaps there is no department of operative surgery, in which there has been the same degree of improvement, within the same space of time, as in the various plastic operations upon different parts of the face, neck, extremities, body, and its cavities.

At the commencement of the present century, autoplasty was confined to the restoration of the nose. Tagliacozzi had demonstrated that deficient portions of the lip might be supplied from the skin of the arm; but it is only during the last fifty years that it has been to any considerable extent resorted to for supplying losses, or congenital defects in other parts of the system. It is at this time not only resorted to advantageously for repairing losses upon the exterior of the body, but cures are obtained in urethral, vaginal, and intestinal perforations, by its interposition. This rapid improvement is due to the researches and experiments of Cooper, Dupuytren, Roux, Lisfanc, Larrey, Velpeau, Lallemand, and Dieffenbach,— and, more especially to M. Jobert. I can have no doubt that Jobert, in his work published in 1821, entitled, "*Traite des maladies voies urinaires*," uttered the unanimous sentiment of the profession, when he announced that "fistulæ, which extend above the neck of the bladder, are incurable." An interesting memoir appeared in 1825, from the pen of M. Lallemand, upon this subject which gave a new impetus to this class of investigations, but which resulted only in producing new modifications of the instrument described in his monograph.

Efforts are made, without much success, to unite the opposing surfaces by pincers, and by the seton, after scarifying the lips of the fistula; and also by the application of potential and actual cauteries to excite the surfaces to union by granulation. In this condition of things, M. Jobert introduced his methods of operating by "*enversement*," and still later, by "*glissement*," (as he terms them,) in vesico vaginal openings, which latter operation has certainly succeeded in curing a proportion of cases of this most loathsome malady Having failed of complete success in the several efforts which I have made after the old method of operating in this intractable disease, and believing the same disappointment to have followed similar attempts made by other American surgeons, I suspect a tolerably minute description of the method by which it is performed by M. Jobert, may not be without interest to yourself, and the readers of the Journal. First, then, in relation to "*enversement*," or *elytroplastie*. In 1834, with a view to a radical cure of this disease, M· Jobert conceived the idea of transplanting a flap of the tissues, more or less remote, into the fistulous opening, and maintaining it there until the union of the opposing surfaces should be complete, thus repairing the loss in the vesico-vaginal partition by a plug or tampon, taken from parts more or less distant. This procedure, which he soon put in execution, consists in simply engrafting one part upon another; and, although M. Jobert may have been the first to make the application, the principle is, after all, but a modification of the old operation upon other parts by torsion, or the *Indian* method as it is sometimes called. The first step in this operation consisted in scarifying the edges of the fistula with scissors, or bistoury, of such form as are best adapted to reach the borders of that under treatment. Then he formed a flap or graft, of such size and form as would fill, without tension, the hole in the vesico-vaginal walls. This was taken from the labia majora, or the thighs, or both, as might be necessary, elevating with the skin the subjacent cellular tissue, so as to preserve the vitality of the part as much as possible, being careful, at the same time, to have the peduncle of such size and length, as to secure the same end, by maintaining the nutrition of the part until union should be accomplished. It is important to the success of the operation that the general health should be good at the time of its performance; for, if the circulation be not vigorously carried on, the transplanted part will be sure to slough. It is unnecessary to enter into a minute description of the manner of forming and dissecting this tampon. Indeed, it must be left to the discretion of the surgeon, after carefully examining all the circumstances pertaining to the case under treatment. Being carefully prepared, it should be gently raised, and twisted into its new situation, when it must be secured by means of sutures. Needles, armed with silk ligatures well waxed, and slightly twisted, are now carried up by means of the port *aiguille*, and passed through the opposing surfaces, nicely adjusted, and temporarily held by long sharp-pointed pincers. It is of the highest importance that each of the angles of the flap should be secured by the sutures, to the corresponding angles of the fistula, otherwise the urine will distil through, leaving a part not united, and making a second operation necessary to perfect the cure. The ligations are then snugly tied, and left until union has taken place, the bladder kept in a state of vacuity meanwhile, by means of a gum elastic catheter, which must be retained in situ with a ribbon secured to an abdominal bandage. The horizontal position must be carefully observed, and the patient kept upon an unstimulating diet until all danger of inflammatory action has passed. It must be remembered, that as the vitality in these parts is much less than in the face or neck, so is union here so much more tardy, aud division of the peduncle must be correspondingly delayed. While in the more vascular region it may ordinarily be divided with safety in four or five days, in the less highly organized parts now under consideration it will often occur that the agglutination is not sufficiently firm to render separation from the parent stock safe before the thirtieth or fortieth day. It is curious to remark the change which immediately succeeds the division of the peduncle in the sensibility of this transplanted tampon. Before its section it retains its connection with the nervous centre, and is sensible to pinching or pricking, but after division these phenomena disappear, and are never recovered. On the contrary, the organic vitality of the transferred surface persists, thus the hairs continue to grow, and have even been found making their appearance at the vulva. Such then is briefly the operation by "*enversement*," as practiced by Jobert for the last fifteen years, if not introduced, as some have hinted, by him in the first instance. It is, however, inapplicable to many cases; for example, if the fistulæ are very large, or high up in the vaginal canal, it is difficult of execution, and the flap is very liable to slough, and the operation prove unsuccessful. Until within the last four or five years, this method of remedying this most uncomfortable affection, with slight modifications suggested by different surgeons, continued to be pursued as the only hope of relief when the fistulæ were of any considerable size. The results of this operation were unsatisfactory to its originator, and he commenced a series of anatomical examinations, which terminated in the conviction that it was possible to repair the loss of substance in the vesico-vaginal partition, by sliding the mucus membrane from the neighboring parts within the vagina itself. This proceeding' M. J. terms, *cystoplastie par glisement,* or *autoplastie vesico-vaginale par locomotion.*

I will endeavor to give you an idea of his manner of proceeding somewhat in detail.

The first step of operation ordinarily consists in prolapsing the uterus to some extent, when the organ has not previously descended from its normal position. It is accomplished in the same manner as when the neck of the uterus is to be extirpated, and one feels some surprise that this method of lessening the distance between the lips of the fistula has not been resorted to by others long before, as it has been well known that the neck of the uterus could be considerably drawn down without danger or much difficulty. The traction must be gradually made by means of strong forceps, of such length as will reach the neck without difficulty, armed at the points with sharp hooks. The little wounds made by the points of the instrument in the neck of the uterus are less likely to be followed by inflammation if seized in a direction opposite to that of the large diameter of the fistule; thus, should the opening in the septum be longitudinal, the forceps should be applied to the sides of the neck; if transverse, the forceps should occupy an antero-posterior direction. This will leave the forceps in a position less likely to interrupt the view of the operator, and it is important that the insertion of the neck of the uterus into the vagina should not be obstructed. The handles of the forceps are now given to assistants, who retain the uterus in this prolapsed state during the operation. Another assistant is placed lower down than the patient who, with a speculum with one valve, and a handle at right angles with the blade, forcibly depresses the perineum at the same time. A semi-circular incision is next made in the vagina, at the point of attachment to the uterine neck, anteriorly and posteriorly, one or both, as may be necessary. This dissection should be made with care, and to such extent as may be requisite to permit sufficient freedom of motion in bringing the edges of the fistula in contact. In making these incisions the bistoury should be kept in contact with the uterine surface, for no danger need be apprehended from wounding its fibres whilst injury to the peritoneum might prove fatal. In making these incisions it is necessary to be familiar with the anatomical relations of the bladder with the uterus anteriorly, and the rectum posteriorly, and the manner in which the peritoneum is connected with these several organs. So, too, is it important to bear in mind, that the relations of that membrane to the bladder varies with its state of distension, and when this organ is permanently contracted, as in fistulæ of long standing, the peritoneum will be still more folded, and dip deeper into the cavity of the pelvis. Now, as the connection between the upper part of the vagina with the organs upon either side is very lax, it is obvious, that by making these incisions at the point of attachment between the uterus and vagina, considerable yielding of the tissues may be expected, and the fistulous surfaces are brought much more readily into contact.

The next step of the operation consists in scarifying, or rather clipping off the edges of the fistula. I am convinced that surgeons have been too timid in their excisions of the edges of fistulæ; in many instances, contenting themselves with simply pairing off the membrane. In fistulæ of considerable duration, the tissues have become indurated, they have lost their vascularity, or vitality, and afford but slight chance of union by the first intention. It is therefore better to secure fresh surfaces from the normal tissue, by cutting away these callous edges. Although a matter of no great moment which lip of the fistula is first scarified, it may be observed, as a good general rule, that it will be best to prepare that lip first, which it is most difficult to bring into view. Thus, when the fistula is transverse, by commencing with the lip nearest the os uteri, which is most difficult of seizure, we shall not be impeded by the hemorrhage which will succeed the cutting off the edges of the lower. In order to effect these excisions, the surgeon may resort to such bistoury, or scissors, as may be best adapted to the fistulæ under treatment. I have seen M. Jobert in the same operation, in removing the indurated edges of a large fistula situated high up in the vagina, have recourse to the straight and curved scissors, to the vaginal bistoury with a sharp and blunt point, in order most effectually and easily to reach its different angles. These surfaces, when low down, may be securely held during this process by the fingers; when near the uterus, long pincers, or hooks with short sharp teeth may be used to bring them into view and retain them there. But whatever instruments may be used matters little, in comparison with the point already insisted upon, that the whole indurated edges be removed, and such healthy surfaces brought together as will unite by first intention.

The surfaces being well prepared, after a little delay, until they shall cease to bleed, should be secured in co-aptation by the interrupted suture. These are inserted by fixing short curved needle sin a *port aiguille* or handle arranged for that purpose, and then, seizing each point of the fistula separately, they should be carried through the vesico-vaginal-substance, from the vagina into the bladder, and then from the bladder into the vagina, and the two ends of the ligature brought out at the vulva. It is important that the entire thickness of the septum be included, and that the points of insertion be so remote from the free edge as not easily to tear out. When the fistula is near the vulvar outlet of the vagina, little difficulty is experienced in carrying the waxed thread, or ligature, through its lips; but when near the uterine extremity it may be difficult, and even impossible to insert the needles in the manner just indicated. To overcome the difficulties incident to a high position of the fistulous orifice, M. Jobert recommends the insertion into the urethra of a sound, armed with a needle, which may be protruded or retracted by means of a handle at its outer extremity. This is carried up to the upper edge of the vesical surface of the fistula, the needle protruded from the sheath, carried through the parieties into the vagina, and one end of the ligature withdrawn, when the needle is retracted into its sheath, brought to the vesical surface of the lower lip of the fistula, and, in like manner, carried into the vagina, through the septum, when the second end of the ligature is disengaged, both being now brought out at the vulva. During these manœuvres the vagina should be protected, and the perineum held back, by an assistant standing to one side, or below the patient, firmly, and holding the speculum of ivory or wood with one valve, already referred to. The points of suture should be four to six times distant from each other, and the ligature itself should be of good size, moderately twisted, and well waxed. Being adjusted as just described, they should be carefully tied, in such manner as merely to bring the opposing surfaces nicely in contact with each other, making moderate pressure only. If too firmly drawn, inflammation of the tissues of the points of the suture may be excited, giving rise to great uneasiness, with frequent desire to micturate. If the ligatures do not tear out in consequence of the swelling, there will be much less probability of union by first intention on account of the incessant contractions of the bladder, and the febrile irritation which will supervene. The vagina may now be carefully freed from any coagula, or fluid blood, and the bleeding restrained by irrigating freely with cold water.

A good gum elastic catheter should now be inserted into the urethra, and permanently retained there by tapes passing from its outer extremity to a bandage around the body. The bladder becomes greatly contracted in old cases of fistula, and is insusceptible of immediate dilatation; besides, the motion occasioned by ultimate dilatation and contraction, would seriously interfere with early union of the parts. Care must be exercised also in selecting the catheter, that it be not too large, else there may be some danger of paralysis of the orbicular muscle, and incontinence in consequence of permanent distension. It must be so placed, if possible, also, that its point be not left in contact with the united surfaces, which might be productive of injurious irritation, and then frequently removed, cleansed, and kept free from obstruction. The coagulation of blood, which may escape for many hours after the operation, might obstruct the flow of urine, unless care were exercised in this respect; and, unless changed frequently, the salts with which this fluid is impregnated, might be deposited about it, and form the nucleus of a calculus. Notwithstanding all these precautions, it will be well, during the first few days which succeed the operation, frequently to percuss the lower part of the abdomen, in order to be perfectly certain that the urine is not retained.

Before inserting the tampon, and ordinarily also before inserting the catheter, after the bleeding had ceased, M. J. carefully examines the approximated surfaces, and, if he finds them tense in every direction, he makes such other divisions of the mucous membrane or vaginal substance, remote from the suture, and in such directions as shall remove all tension. One is surprised to see with what impunity these incisions are made, and not less remarkable is it to observe the facility with which these parts slide towards the fistula, and relax the tension upon the ligatures. These incisions are ordinarily made with a long bistoury in the vaginal membrane, in a direction parallel to the suture ; but, when necessary to produce perfect relaxation of parts, I have seen M. J. divide in different directions the tissues quite external to the outlet of the canal.

The vagina being again carefully freed from blood, is filled by M. J. with a tampon, *d'amadou*, or tinder, or spunk, as it is called in common American parlance, of such size as can be inserted without inconvenience. The object in placing this in contact with the recently-incised surfaces, is to absorb any blood which may discharge from them during the few hours which succeed the operation. It should be so placed that it may be extracted without danger of tearing open the sutures. It should be removed in the course of the next day by gentle tractions upon a ligature attached to its lower extremity, before putting it in place. The patient should now be carefully placed in bed, where she should observe the recumbent position for several days, lying, as much as possible, upon the back. During the succeeding week, her diet should be light, easy of digestion, and such as will not be likely to excite the bowels to action, or generate gas. And here I may remark, that the patient should be prepared for the operation much as for those upon the rectum. It is perceived that this method of procedure is not demanded in cases of traumatic fistula, such as simple incisions, or when the loss of substance is inconsiderable from any cause; but it would seem to be peculiarly adapted to the most common form of this affection, which being ordinarily the result of protracted pressure during labor, and in which the amount of substance lost from the sloughing which follows, is very great. When the fistula results from this cause, whilst an early operation is always desirable, for many reasons, some care must be taken not to undertake any procedure whilst the female remains in the puerperal state, nor until tone is restored in some degree to the edges of the fistula. It is well known that for some time after accouchment, the patient is much more liable to a variety of affections; and all wounds, according to M. J., made at this period, are peculiarly prone to suppuration. He recommends, however, that a catheter be immediately introduced into the bladder, and retained there until the proper period for operating arrives, for the purpose, not only of conducting the urine through the natural channel, but to accustom the mucous membrane of the bladder to the presence of the instrument, so that it may be borne with less irritation when its presence shall be necessary. He further suggests, that it is best to make the operation just after the menses have ceased, and avoid the danger to which the menstrual molimen would expose the patient.

An interesting inquiry very naturally suggests itself relative to the effect which the free incisions made in the vagina, in this operation, produce upon its tissues. In the cases which I have witnessed when the operation had been made some months before, there were to be seen simply eschars, more or less distinct, at the point where the fistula had been closed at the anterior surface of the neck of the uterus, and such other points as had been incised. These were not indurated, and the membrane in the immediate vicinity lay in folds, leaving, in all respects, a natural appearance. M. Jobert assures us that the canal is not weakened by this operation, and that children pass through it with as much safety as before; that the bladder, which has become very much contracted, gradually expands, and eventually retains the urine as before the existence of the malady; that the uterus ordinarily remains slightly prolapsed; that the parieties of the vagina regain their firmness, and the canal retains its normal direction, the point of connection with the canal only being slightly changed. The uterus, which has had its functions more or less impaired by the presence of the urine, rapidly resumes its normal condition; the general health, which always suffers more or less in chronic fistula, is rapidly restored, and, more than all, the spirits, which are ordinarily depressed, become cheerful, and hope and buoyancy take the place of despondency.

There are unfortunately some cases of fistula in which no operation would avail, such as cancer of the uterus and vagina, which often destroys this partition long before death. It is obvious, also, that the destruction of the septum by sloughing after parturition, may be so extensive as to exclude all hope of reparation by any operation, although M. Jobert insists that the loss of the urethra is not an obstacle to complete cure.

Whether this operation will fulfill the expectations of its inventor, is perhaps still somewhat problematical. I have witnessed six operations by M. Jobert, and followed them to their conclusion. Two of these were entirely successful, the union being complete by the first intention. Two more were greatly improved, the fistulæ greatly diminished in size, but still leaving an angle ununited, which will require the use of some caustic for some time, or another slight operation to perfect the cure. A fifth patient, of bad constitution, and in feeble condition at the time, was scarcely improved, the edges of the fistula did not unite, though very likely attributable to the vitiated state of her general health. The sixth died soon after the operation, with symptoms of peritonitis; but whether it was mere coincidence, as was claimed, or the result of inflammation excited by wounding the peritoneum, as was hinted, I am unable to determine. Not expecting so abrupt a conclusion, I did not see the patient after the operation. Were such an accident to occur in private practice in America, after an operation which was not demanded for the preservation of life, it would be very likely to bring it into disrepute. Irrespective of the fatal case, there would be every reason to conclude that a great triumph, in the cause of humanity and science, had been achieved. If it can be resorted to without danger to life, though it might not succeed in more than one-half the subjects, provided their condition was not prejudiced, it would certainly be a great blessing in a class of maladies so loathsome, so destructive of happiness, and rendering the patient so disgusting to herself and family, that relief by death is often heartily welcomed by its miserable victims.

For the present, I can only say, that I intend to pursue the subject farther; and, should any satisfactory conclusions be the result of such an investigation, you will be subjected to another letter. Meantime, regretting that I have allowed my own interest in this matter to lead to so long an infliction upon yourself,

I remain, your friend,

JAMES P. WHITE.

